# The relationship between negative life events and cortical structural connectivity in adolescents

**DOI:** 10.1016/j.ibneur.2024.01.012

**Published:** 2024-02-01

**Authors:** Francesca Sibilia, Coline Jost-Mousseau, Tobias Banaschewski, Gareth J. Barker, Christian Büchel, Sylvane Desrivières, Herta Flor, Antoine Grigis, Hugh Garavan, Penny Gowland, Andreas Heinz, Bernd Ittermann, Jean-Luc Martinot, Marie-Laure Paillère Martinot, Eric Artiges, Frauke Nees, Dimitri Papadopoulos Orfanos, Luise Poustka, Sabina Millenet, Juliane H. Fröhner, Michael N. Smolka, Henrik Walter, Robert Whelan, Gunter Schumann, Arun L.W. Bokde, IMAGEN Consortium

**Affiliations:** a Discipline of Psychiatry, School of Medicine and Trinity College Institute of Neuroscience, Trinity College Dublin, Dublin, Ireland; b Paris Institute of Technology for Life, Food and Environmental Sciences, Paris, France; c Department of Child and Adolescent Psychiatry and Psychotherapy, Central Institute of Mental Health, Medical Faculty Mannheim, Heidelberg University, Square J5, 68159 Mannheim, Germany; d Department of Neuroimaging, Institute of Psychiatry, Psychology & Neuroscience, King’s College London, United Kingdom; e University Medical Centre Hamburg-Eppendorf, House W34, 3.OG, Martinistr. 52, 20246, Hamburg, Germany; f Medical Research Council - Social, Genetic and Developmental Psychiatry Centre, Institute of Psychiatry, Psychology & Neuroscience, King’s College London, United Kingdom; g Department of Cognitive and Clinical Neuroscience, Central Institute of Mental Health, Medical Faculty Mannheim, Heidelberg University, Square J5, Mannheim, Germany; h Department of Psychology, School of Social Sciences, University of Mannheim, 68131 Mannheim, Germany; i NeuroSpin, CEA, Université Paris-Saclay, F-91191 Gif-sur-Yvette, France; j Departments of Psychiatry and Psychology, University of Vermont, 05405 Burlington, VT, USA; k Sir Peter Mansfield Imaging Centre School of Physics and Astronomy, University of Nottingham, University Park, Nottingham, United Kingdom; l Charité – Universitätsmedizin Berlin, Department of Psychiatry and Psychotherapy, Campus Charité Mitte, Charitéplatz 1, Berlin, Germany; m Physikalisch-Technische Bundesanstalt (PTB), Braunschweig and Berlin, Germany; n Institut National de la Santé et de la Recherche Médicale, INSERM Unit 1000 “Neuroimaging & Psychiatry”, University Paris Saclay, University Paris Descartes – Sorbonne Paris Cité; and Maison de Solenn, Paris, France; o Institut National de la Santé et de la Recherche Médicale, INSERM Unit 1000 “Neuroimaging & Psychiatry”, University Paris Saclay, University Paris Descartes; and AP-HP.Sorbonne Université, Department of Child and Adolescent Psychiatry, Pitié-Salpêtrière Hospital, Paris, France; p Institut National de la Santé et de la Recherche Médicale, INSERM Unit 1000 “Neuroimaging & Psychiatry”, University Paris Sud, University Paris Descartes - Sorbonne Paris Cité; and Psychiatry Department 91G16, Orsay Hospital, France; q Department of Child and Adolescent Psychiatry and Psychotherapy, University Medical Centre Göttingen, von-Siebold-Str. 5, 37075, Göttingen, Germany; r Department of Psychiatry and Neuroimaging Center, Technische Universität Dresden, Dresden, Germany; s School of Psychology and Global Brain Health Institute, Trinity College Dublin, Ireland

**Keywords:** Graph theory, Brain networks, Cortex, Adolescence, Stress, Edge connectivity

## Abstract

Adolescence is a crucial period for physical and psychological development. The impact of negative life events represents a risk factor for the onset of neuropsychiatric disorders. This study aims to investigate the relationship between negative life events and structural brain connectivity, considering both graph theory and connectivity strength. A group (n = 487) of adolescents from the IMAGEN Consortium was divided into Low and High Stress groups. Brain networks were extracted at an individual level, based on morphological similarity between grey matter regions with regions defined using an atlas-based region of interest (ROI) approach. Between-group comparisons were performed with global and local graph theory measures in a range of sparsity levels. The analysis was also performed in a larger sample of adolescents (n = 976) to examine linear correlations between stress level and network measures. Connectivity strength differences were investigated with network-based statistics. Negative life events were not found to be a factor influencing global network measures at any sparsity level. At local network level, between-group differences were found in centrality measures of the left somato-motor network (a decrease of betweenness centrality was seen at sparsity 5%), of the bilateral central visual and the left dorsal attention network (increase of degree at sparsity 10% at sparsity 30% respectively). Network-based statistics analysis showed an increase in connectivity strength in the High stress group in edges connecting the dorsal attention, limbic and salience networks. This study suggests negative life events alone do not alter structural connectivity globally, but they are associated to connectivity properties in areas involved in emotion and attention.

## 1 Introduction

Adolescence is defined as a transitional period between childhood and adulthood, with physical, emotional and physiological changes. It begins with puberty until the attainment of sexual maturity and neurobehavioral characteristics associated with the adulthood (Holmbeck, 2002). Throughout adolescence, brain changes occur in both grey (GM) and white matter (WM), reflected in the structural re-organization of affective and cognitive systems (Juraska and Markham, 2004, Scherf et al., 2013).

Giedd and colleagues described how GM volume changes follow an inverted U shape from childhood to adulthood (Giedd et al., 1999), reaching its peak during adolescence. In particular, the frontal and parietal lobes reach the peak sooner than the temporal lobe (Gogtay et al., 2004). Evans described connectivity covariance of morphological metrics based on structural imaging throughout life (Evans, 2013), examining the development of brain networks from childhood to adolescence. They showed that local efficiency of neural communication among brain regions increased until late adolescence, while global efficiency showed the opposite trend. When they explored region-specific changes, they found within- and between-connectivity increases in limbic and association regions during the later ages of adolescence (Khundrakpam et al., 2013).

The high level of plasticity characterizing adolescence makes the brain sensitive to stress following negative events, which may influence the typical developmental pathway. The term “stress” was coined by Hans Selye in 1936 to indicate “the non-specific response of the body to any demand for change” (Szabo et al., 2012), whereas the event triggering such response was defined “stressor”. There are many studies showing an association between stress and neural network interactions, and there has been a particular attention on amygdala connectivity (Etkin et al., 2011, Hakamata et al., 2017, Mehta et al., 2018) which are linked to alterations in hypothalamic-pituitary-adrenal (HPA) axis function (Urry et al.,, Gee et al., 2013). Such alterations can affect the efficiency of information flow among brain regions, especially in limbic areas associated to decision making, emotion and reward systems (Eiland and Romeo, 2013). Previously it has been shown that the severity and nature of negative events affects different areas of the adolescent brain: adverse early-life events were associated with decreases of grey matter volume in the anterior prefrontal areas, whereas socially stressful events were related to changes in orbitofrontal cortex, amygdala and other subcortical regions (Tyborowska et al., 2018).

The impact of adverse early-life events on the brain has been associated to mnemonic and emotional processes of the limbic system (Khundrakpam et al., 2013). One study investigating the relationship between perceived stress and emotional-related brain networks showed how the amygdala and the ventromedial prefrontal cortex interaction changes at different stages of adolescence, suggesting a different emotional reaction to stress over time. They found a positive relationship between perceived stress and amygdalar-ventromedial prefrontal connectivity in adolescence, whereas young adults showed the opposite pattern (Wu et al., 2018). Other studies showed connectivity alterations in adolescents who suffered maltreatment (Ohashi et al., 2017), PTSD (Suo et al., 2015) and MDD (Xu et al., 2018), especially in the salience, default-mode (DMN) and frontolimbic networks (McEwen, 2012); disrupted connectivity associated to adverse events can affect aspects of self-awareness and introspection crucial during this life stage. Regarding the brain regions more affected by stressful events, those found to be altered were the middle temporal gyrus, dorsolateral prefrontal, posterior cingulate and occipital cortices, when compared to healthy controls (Viard et al., 2019).

Other studies have focused on the effect of stress on the functional connectivity in resting state networks, for example it has been found that adolescents that experienced childhood abuse had lower functional connectivity between the left prefrontal cortex to other regions within the frontal-parietal network (FPN) during performance of a demanding attentional task compared to healthy subjects (Hart et al., 2017). Another study that examined the same brain network, found that psychosocial stress was correlated to impaired attentional control and a decrease in functional connectivity within the FPN (Liston et al., 2009). The results related to the FPN are consistent with the idea that stress are associated with decreases in functional connectivity within the FPN, as well as decrease performance during attentional tasks mediated by the FPN network.

Beyond the FPN network, there have been cross-sectional studies examining the impact of early adversities have found changes in fMRI resting state networks in the default mode network (DMN), salience network (SN), dorsal attentional network (DAN) (Herzberg et al., 2021, Fan et al., 2017, Wang et al., 2014). The effect of stress, quantified using graph analysis, have found differences based on resting state data in the context of childhood abuse (Wozniak et al., 2013), maternal separation (Herzberg et al., 2021), and prenatal alcohol exposure (Long et al., 2019). In addition, a longitudinal study of adolescents that were exposed to childhood maltreatment, found maltreatment to be associated with increased between-network connectivity (Rakesh et al., 2021). Another longitudinal study, including adolescents from ages 9 to 19, found altered developmental network plasticity (SAN, DMN, FPN) as a function of deprivation, neglect, and unpredictability, with widespread brain changes in response to deprivation but the effects of unpredictability localised to the SAN (Chahal et al., 2022). The cross-sectional and longitudinal studies show that psychosocial stress impacts the developmental pathway of the brain although there are inconsistencies across studies that may be related to differences in recruited groups, measurements differences (of stress) across studies, as well as differences in neuroimaging analysis stategies.

In addition to functional connectivity, structural connectivity measures have been shown to be a valid biomarker to investigate stress effects on the adolescent brain (Khundrakpam et al., 2016). Graph theory can be used to calculate connectivity measures (Sporns, 2013) that quantify structural network properties of centrality, segregation and integration between different “nodes” (brain regions) connected by “edges” (structural connections between nodes) (Bullmore and Sporns, 2009, van den Heuvel and Sporns, 2013).

The objective of this study is to explore differences in structural connectivity strength and brain network properties in a population of healthy adolescents with adverse life events. In the first analysis, we examined if there were differences in structural connectivity between two groups of adolescents that reported different levels of perceived early-stress life events. In a second analysis we examined if there was a linear association between the number of stressful events and changes in structural connectivity.

## 2 Methods and materials

### 2.1 IMAGEN population

Participants were from the IMAGEN study (Schumann et al., 2010), a longitudinal study on adolescent brain development and behavior, with data being collected from eight different centers across Europe (in Germany, UK, France and Ireland). The IMAGEN cohort has more than 2200 adolescents who underwent a series of behavioral, neuropsychological assessments, genetic screening and neuroimaging. Parents gave informed written consent and adolescents gave written assent to the study procedure prior to inclusion. All procedures were approved by each local institutional ethics committee. Further descriptions of the study design, sample, and recruitment procedure, including data storage and safety can be found elsewhere (Schumann et al., 2010). Table 1, Table 2 describe the demographic information of the sample used for each analysis, represented by participants who passed the MRI quality check and had all the covariates included in the analysis. The Pubertal Development Scale (PDS) is a commonly used self-report questionnaire designed to assess the stage of pubertal development in adolescents. The Socio-Economic-Status (SES) scores describe the occupational status and educational attainment of the participants’ parents; the total SES score is obtained by summing the numerical values assigned to each category of parental occupation and education. The SES is considered to be an important factor influencing psychological outcomes and provides a measure of the participant’s access to economic resources and social position in relation to others. To assess for the potential presence of mental health disorder, the participants were assessed with the Development and Well-Being Assessment (DAWBA) questionnaire (Goodman et al., 2000), which is designed to generate DSV-IV and ICD-10 psychiatric diagnosis – in our sample the mean probability of diagnosis of depression, general anxiety or post-traumatic stress disorder were very low (see Table 1).

**Table 1:** Demographic information of the sub-sample considered in this study. Abbreviations: NLEs = negative life events; LEQ= life event questionnaire; PDS= pubertal development scale; SES= socio-economic status.

N = 487	Low Stress		High Stress		p-value
Group size	360		127		
Gender	Female	178 (49.4%)	Female	72 (56.7%)	p = 0.160
	Male	182 (50.6%)	Male	55 (43.3%)	
Age	14.44 ± 0.56		14.47 ± 0.547		p = 0.527
Nr of NLEs	2.71 ± 1.22		8.96 ± 1.41		p < 0.001
LEQ scores	-3.36 ± 1.419		-14.18 ± 2.389		p < 0.001
PDS	15.89 ± 2.830		16.68 ± 2.439		p = 0.99
SES	6.76 ± 3.215		8.5 ± 3.172		p < 0.001
DAWBA	Depression	0.32 ± 0.65	0.61 ± 0.88		p < 0.001
	Anxiety	0.29 ± 0.67	0.78 ± 1.06		p < 0.001
	Post-traumatic stress	0.01 ± 0.10	0.08 ± 0.34		p < 0.001

**Table 2:** Demographic information of the sample considered in the partial correlation analysis. Abbreviations: NLEs = negative life events; LEQ= life event questionnaire; PDS= pubertal development scale.

**N = 976**		
Gender	Male	452 (46%)
	Female	524 (54%)
Age	14.45 ± 0.453	
Nr of NLEs	5.09 ± 2.465	
LEQ score	-7.199 ± 3.9	
PDS	16.29 ± 2.72	

### 2.2 Participants for between-group comparison

There were 487 participants (age mean=14.45 ± 0.55) divided into Low Stress and High Stress groups (see Table 1 for demographic data). In a previous study, participants were defined to have high levels of stress when they experienced NLEs ≥ 4, which was shown to be a statistically significant exposure to stress in 15% of the population (Galinowski et al., 2015) (Caspi et al., 2003). The number of NLEs and the cumulative negative score were used as cut-off value to divide participants into Low and High stress groups. In this study, adolescents who experienced from none to 5 NLEs with a total score from − 5 to 0 were categorized as Low Stress, while those who had 6 or more NLEs and total score from − 20 to − 11 were defined as High Stress. Both groups were gender balanced (p = 0.16).

### 2.3 Negative life events assessment

The perceived stress levels due to negative life events were measured with the Life Event Questionnaire (LEQ) (Newcomb et al., 1981), - a 39-item questionnaire – which was used to assess the level of stress in participants, with each question describing a life event, and participants were asked to indicate if such event ever happened in their lifetime. The questions from the LEQ used in this study are based on those chosen in a previous study by Galinowski (Galinowski et al., 2015) plus two more, i.e., “changed school” and “got poor grades at school”. The scoring scale for each question had a range from − 2 (very negative) to + 2 (very positive), indicating the event desirability. The number of total NLEs was calculated by adding up every event rated as negative by the participant.

### 2.4 Imaging

The magnetic resonance imaging (MRI) scans were acquired at eight IMAGEN sites with all centers using 3 T MRI systems. High-resolution T1-weighted images were obtained from different manufacturers (Siemens: five sites, Philips: two sites, General Electric: one site). Briefly, T1-weighted anatomical images were acquired using 3D MPRAGE sequences (resolution = 1.1 ×1.1 ×1.1 mm; TR = 2300 ms; TE = 2.9 ms). Functional images were acquired with GE-EPI sequences (resolution = 3.4 × 3.4 mm; slice thickness = 2.4 mm; TR = 2200 ms; TE = 30 ms). MRI acquisition protocols and quality checks have been described elsewhere (Schumann et al., 2010), based on the ADNI protocol (http://www.loni.ucla.edu/ADNI/Cores/in-dex.shtml), which allowed comparable data to be acquired from all sites despite these scanner differences. More details are available on the IMAGEN github page: https://github.com/imagen2/imagen_mri.

### 2.5 MRI Analysis

#### 2.5.1 Preprocessing and Extraction of Brain Networks

Images were pre-processed in SPM8, and segmented into grey matter (GM), white matter (WM) and cerebro-spinal fluid (CSF). Brain networks were extracted using a method previously published (Tijms et al., 2012). Briefly described, the technique divides the gray matter segmentation of an individual's brain from the first nonempty voxel into cubes with dimensions of 3x3x3 voxels (6x6x6 mm^3^). Morphological similarity between all cube pairs (ROIs) was calculated based on linear correlation (Tijms et al., 2013), rotating each cube relative to the other one to find the maximum linear correlation between them (this was the final correlation coefficient value considered for each voxel pair). The utilization of these cubes preserves the 3D integrity of the cortex, leveraging spatial details from the MRI scan alongside the gray matter voxel values. This preservation of spatial information within the cubes yields a parameter that mirrors the local thickness and folding pattern of the cortex (Tijms et al., 2013). Previously, structural connectivity calculated from cortical thickness was used to describe the modular architecture of the brain based on the morphological connections between different regions (Chen et al., 2008). Such approach has been used for group-averaged network analysis, while the focus of this study was to investigate connectivity differences on a single-subject level (for more information about brain morphology: (Madan, 2017)).

#### 2.5.2 Matrix resizing

The atlas used for the voxel-based analysis was developed by Schaefer and colleagues, organized into 400 ROIs and 17 networks (Schaefer et al., 2017). Table 3 describes the 17 networks and the brain areas that are part of each network. A complete list of the 400 parcels belonging to the 17 networks is found in the Supplementary Methods and in Figs. S1 and S2.

**Table 3:** Regions of interest (ROIs) that comprise the 17 networks in Schaefer template. Additional details of the networks are included in the supplementary data.

**Networks**	**Name network**	**ROls**
1	Visual Central	Striate, extrastriate
2	Visual peripheral	Striate, extrastriate
3	SomatomotorA	Central sulcus, secondary somatosensory
4	SomatomotorB	Auditory cortex, central sulcus
5	DorsaI Attention A	Temporal Occipital, parietal occipital, superior parietal lobule
6	Dorsal Attention B	Post-cingulate, frontal eye field, precuneus
7	Salience A	Ventral Attention Par opercularis, insular cortex, medial parietal and frontal
8	Salience B	Ventral Attention Lateral, ventral, medio-parietal, orbital frontal cortex
9	Limbic	Orbito-frontal
10	Limbic	Temporal pole
11	Control A	Temporal, inferior parietal sulcus, dorsal and lateral prefrontal cortex, cingulate
12	Control B	Inferior parietal lateral, dorsal, ventro-lateral prefrontal and medio-parietal frontal
13	Control C	Posterior cingulum, precuneus
14	Default A	Dorsal and medio-dorsal prefrontal, posterior cingulate
15	Default B	Temporal, inferior parietal, ventral and dorsal prefrontal
16	Default C	Inferior parietal, retrosplenial, parahippocampal
17	Temporo-parietal	Temporal cortex

The atlas was non-linearly registered to individual MRIs using DARTEL in SPM8 (Klein et al., 2009) to adjust for the different brain shape across participants. The linear correlation coefficient between each pair of voxels was allocated to the respective 400 ROIs in the atlas. All the linear correlation values belonging to the same ROI were then averaged, obtaining a matrix of 400 × 400 for each participant – we defined this process matrix resizing. To create a symmetric matrix, the transpose of the upper triangle was calculated. A graphical representation of the correlation matrices resizing is included in the Supplementary Material (Fig. S3).

#### 2.5.3 Thresholding levels

**Fig. 1:**
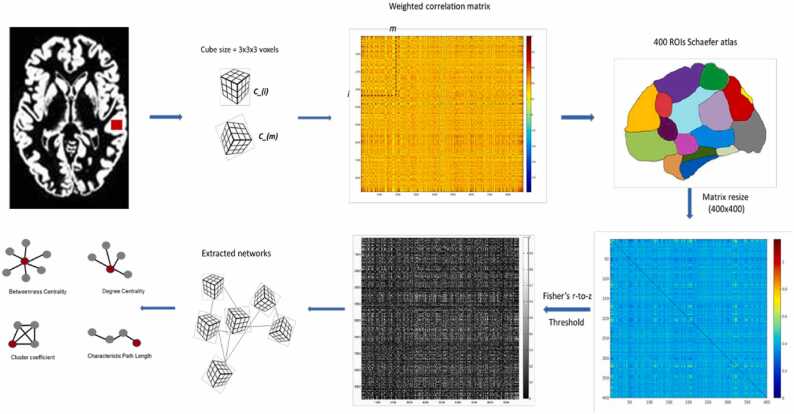
Brain network extraction pipeline**.** Graphical representation of the method used to calculate brain networks and extract graph theory measures. Correlation matrices are built by calculating the morphological similarity between two cubes. Connectivity matrices are resized based on an atlas with 400 ROIs, and binary matrices are produced applying different thresholds which reflect different sparsity levels.

Fisher’s r-to-z transformation was applied to the correlation matrices. Seven different sparsity levels (from 0.05 to 0.35 with increments of 0.05) were chosen to investigate whether different thresholds affected the network analysis between groups. Sparsity is defined as connectivity density in brain networks, i.e., the percentage of existing connections compared to the maximum number of possible connections in the network (Sporns, 2013). For example, with a sparsity level of 0.2, only the highest 20% of all the connections was retained to calculate the graph measures. Finally, all the correlation matrices were binarized to create unweighted and undirected networks (see Fig. 1).

Graph theory measures were quantified using Brain Connectivity Toolbox (Rubinov and Sporns, 2010). The global graph theory measures calculated were mean cluster coefficient (CP), mean degree centrality (DC), characteristic path length (LP), small-worldness (SW) and global efficiency. The local graph theory measures were nodal betweenness centrality (BC), nodal degree and nodal clustering coefficient. Cluster coefficient (CP) describes the number of connections among a node's topological neighbors (Sporns, 2013), and it can provide insights into the local efficiency and organization of neural connections. Characteristic path length (LP) indicates the global average of the shortest paths in the network (Rubinov and Sporns, 2010) and it is linked to global efficiency of information processing, that is the average of the inverse of all the distances across the nodes (Stam and Reijneveld, 2007). Node centrality is measured by degree and betweenness centrality (i.e., how central a brain region is within a network compared to others). Degree centrality (DC) is defined as the number of edges for each node (Bullmore and Sporns, 2009, Rubinov and Sporns, 2010), while betweenness centrality (BC) measures centrality at a local level, indicating the fraction of all shortest paths in the network that pass through a given node (Stam and Reijneveld, 2007). Nodes with high BC or DC are defined network hubs (van den Heuvel and Sporns, 2013). Hub nodes may play a crucial role in integrating information and facilitating communication between different parts of the brain. Networks with high CP and short LP present a property of small-worldness, which influences the network wiring cost (Watts and Strogatz, 1998).

### 2.6 Statistical analysis

#### 2.6.1 Between-group comparisons

To examine between-group statistical differences on global graph theory measures, ANCOVA models (in SPSS v.24, IBM Inc, USA) were used on the mean cluster coefficient, mean degree centrality, path length, global efficiency and small-worldness, with age, sex, center, PDS and social-economic score (SES) as covariates. Sex and center were dummy coded in the model. Included in our statistical models were age, sex, center, pubertal development scale (PDS) and SES as covariates.

At a local level, only network hubs were inspected, running two-tailed t–tests on nodal betweenness and degree centrality and cluster coefficient. In this study, ROIs which had BC and DC values at 2 SD above the mean were identified as network hubs. For the CP all the 400 ROIs were considered, since differences in overall distributions between the groups were of interest. Statistical tests were corrected for multiple comparisons using false discovery rate (FDR), based on the Benjamini and Hochberg procedure (Benjamini and Hochberg, 1995).

#### 2.6.2 Whole-group correlation between graph theory and stress measure

We investigated if there was a linear association between the global graph theory measures (mean CP, LP, SW, global efficiency and mean DC) and the level of stress for each participant (represented by the total number of NLEs individually). The partial correlation coefficient was calculated at each sparsity level controlling for age, gender, center and PDS (Petersen et al., 1988). PDS scores were standardized, using z-transformation.

#### 2.6.3 Connectivity strength analysis with NBS

Our interest was also investigating the association between stress with edge connectivity strength between two nodes (such strength is indicated graphically by different edge thickness). To do so, we used Network-based Statistics (NBS) (Zalesky et al., 2010), a toolbox based on a nonparametric statistical method to correct for multiple comparisons. In this study between-group differences at a single connection level were analyzed, controlling for false discovery rate, based on 100,000 permutations, α = 0.05 and a two-sided t-test between groups. Any edge showing a p-values lower than the α value was considered statistically significant.

## 3 Results

### 3.1 Between-group differences

Chi-square tests showed no statistically significant differences between groups in age, sex, or PDS scores, and the two groups did not differ in intracranial volume (p = 0.23). There was no statistically significant effect of scanning centers when comparing between groups (χ² = 0.104).

There were no statistically significant between-group differences in the global graph theory measures. The statistical model was run at each sparsity level both with and without SES (SES was statistically significantly different between the two groups); inclusion of the SES covariate in model did not alter results. Confidence intervals (CIs) at 95% for the global measures at each sparsity level are shown in Fig. S4. The data were also analysed with inclusion of the intracranial volume as a covariate – no changes in results from previous model were found.

**Fig. 2:**
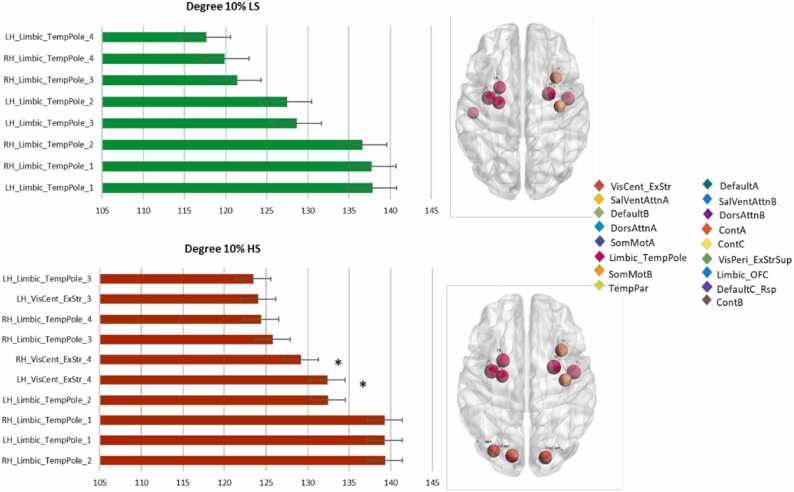
**Between-group degree centrality differences**. Hubs rank changes between Low and High stress groups based on degree centrality at sparsity of 10%. The ROI names on the *y* axis indicate the hemisphere, the network, the brain area and the number of the brain area subregions. The size of the node is proportional to betweenness value and color of node indicates to which neural network it belongs to. Starts are indicating statistically significant differences of degree centrality between groups. Error bars indicate the standard error.

Fig. 2 shows the node rank in the two groups at sparsity 10% based on DC. Statistically significant increases of DC were found in the High Stress group in the left (xyz coord: [−14 −84 −13]) and right (xyz coord: [18–86]) extrastriate areas of the central visual network at sparsity 10% (t = −2.736, p_corr_=0.048 and t = −2.951, p_corr_= 0.048 respectively), and in the posterior cingulate node of the left dorsal attention network (xyz coord: [−42 −37 46]) at sparsity 30% (t = −2.993, p_corr_= 0.042). No between-group statistically significant differences were detected in the other sparsity levels.

**Fig. 3:**
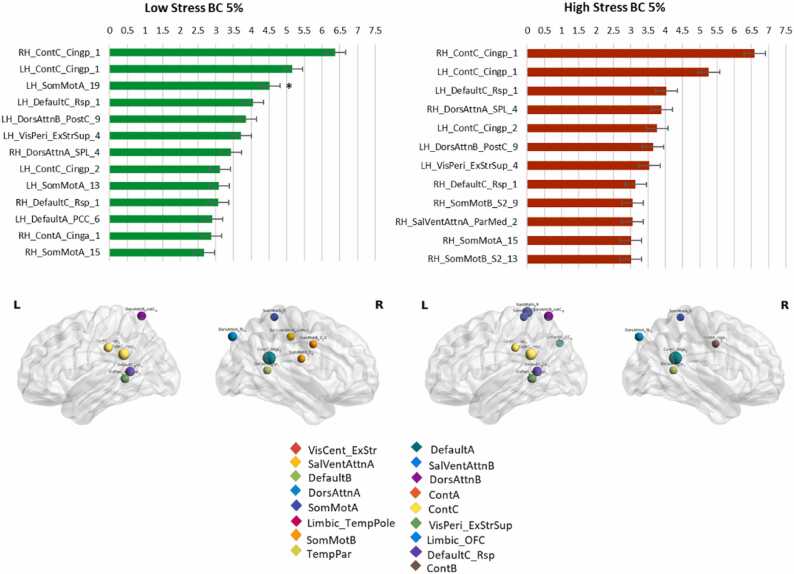
**Between-group betweenness centrality differences.** Hubs rank in both groups based on betweenness centrality at sparsity= 0.05. In the bottom part of the figure, the size of the node is proportional to betweenness value and color of node indicates to which neural network it belongs to. In the bar charts, the star indicates the region with a statistically significant between-group difference (FDR corrected). The ROI names on the *y* axis indicate the hemisphere, the network, the brain area and the number of the brain area subregions. Error bars indicate the standard error.

At a sparsity 5%, there was a statistically significant decrease of BC in the left somato-motor cortex (xyz coord: [−10 −41 72]) in the High stress group (t = 3.674, p_corr_=0.0042). Fig. 3 also illustrates the location of the highest nodes at this sparsity level. No other statistically significant differences between BC hubs were detected in other sparsity levels.

Examining nodal CP, where all 400 nodes were considered, no p-value survived FDR correction, except for the network at sparsity 35%, where an increase was seen in part of the orbito-frontal cortex (p_corr_= 0.04) in the High stress group. No statistically significant between-group differences were seen at the other sparsity levels.

### 3.2 Whole-group partial correlations

Partial linear correlations showed no statistically significant linear correlation between any of the global measures and level of stress in the whole group of adolescents (p > 0.05). A graphical example of partial linear correlation plots at sparsity 15% is shown in Fig. S5.

### 3.3 Connectivity strength results

**Fig. 4:**
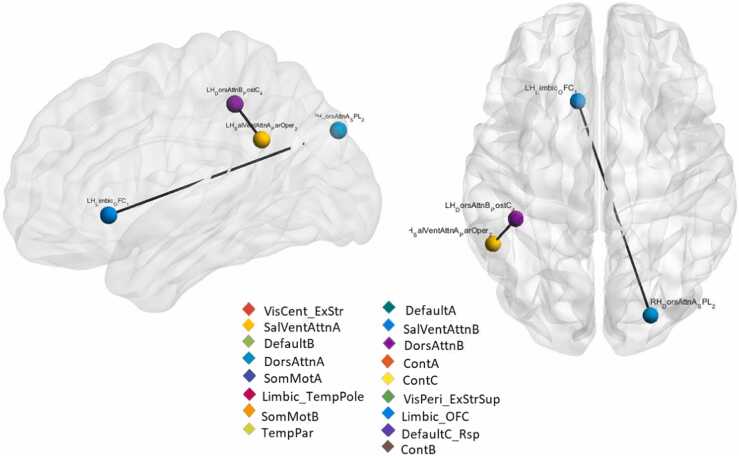
Between-group connectivity strength differences. Single edges showing statistically significant increase of connectivity in High stress group compared to Low stress group. The ROI names indicate the hemisphere, the network, the brain area, and the number of the brain area subregions.

There were statistically significant increases of edge connectivity strength in the High stress compared to the Low stress as shown in Fig. 4. Specifically, two statistically significant increases were seen: an inter-hemispheric edge connecting a part of the orbito-frontal cortex (xyz coordinates = [−11 21 −4]) to part of the right superior parietal lobule (xyz coord= [25 −85 34]), belonging respectively to the limbic network and dorsal-attention network (t-value= 3.83, p < 0.0001). The second edge connected the posterior cingulate cortex node of the left dorsal attention network (xyz coord= [−42 −37 46]) to the pars opercularis node of the left salience network (xyz coord= [−53 −49 30]), t-test = 4.39, p < 0.00001. There were no statistically significant increases in connectivity detected in the Low stress compared to the High stress.

## 4 Discussion

In this study we examined if there were structural connectivity alterations with negative life events in the adolescent brain. Graph theory measures were calculated across a range of different sparsity levels. In the between-group analysis, there were no changes in global network measures between Low and High Stress groups. The results from local measures analyses showed differences in centrality measures only at specific thresholds, otherwise no statistically significant results were detected between groups. In addition, the analysis on connectivity strength with NBS revealed hyper-connectivity between edges connecting nodes that belonged to emotion and attention-related networks.

The lack of statistically significant results in the global measures between groups suggested that stressful early-life events does not lead to global structural connectivity changes in the brain. Ho and colleagues reviewed studies on stress effect on brain connectivity, considering both graph theory metrics and edge strength, and found no statistically significant differences in global graph theory measures between adolescents suffering of neuropsychiatric disorders and healthy controls. They concluded this might be because the neurobiological basis of such metrics such as small-worldness, path length and efficiency is well established before adolescence (Ho et al., 2018).

In the second analysis, we examined if there was a linear association between number of stressful events and brain connectivity. The lack of statistically significant relationship between the LEQ and the global network measures across sparsity levels has been confirmed by previous findings (Ho et al., 2018), showing that stress due to negative life events is not associated to changes of global connectivity in adolescents. A few of studies suggested that employing diverse stress measures and considering varying severity levels may result in unique brain responses, as evidenced by specific patterns of connectivity. The implication is that the choice of stress assessment and its intensity can influence the observed neural connectivity patterns, emphasizing the importance of considering these factors in the interpretation of study outcomes (van Oort et al., 2017, Keshmiri, 2020).

The connectivity strength analysis with NBS showed an increase in two edges of the High stress group. The first edge connected the orbito-frontal cortex (OFC) of the left limbic network to the superior parietal lobule (SPL) of the right dorsal attention network (DAN). The second edge connected the pars opercularis of the left ventral attention network (VAN) to the posterior cingulate cortex of the left DAN. These results confirmed previous studies on connectivity, showing the role of SPL in top-down attention and visuospatial cues processing (De Bellis et al., 2015); volume reductions in this region were found in adolescents suffering with stress-related disorders, for example PTSD (Tan et al., 2013) and MDD (De Bellis et al., 2015). In another study structural alterations in the OFC and parietal lobe were found in children victim of physical abuse, correlated with social and learning difficulties (Hanson et al., 2010).

The pars opercularis within the cingulo-opercular network (CON), plays a role in maintaining vigilance and alertness for tasks involving working memory (Sadaghiani and D'Esposito, 2015). This node has shown a strong responsiveness to cortisol levels in the body (Frodl and O'Keane, 2013), and alterations in its connectivity have been linked to both general and social anxiety disorders (Sylvester et al., 2012). Sheth and colleagues suggested chronic stress might lead to disruption between limbic and prefrontal circuits during adolescence. Alterations in the connectivity of the ventromedial prefrontal and orbitofrontal cortex have been observed in correlation with elevated cumulative stress. This is linked to increased propensity for risk-taking behaviors and a reduction in the regulation of emotional responses (Sheth et al., 2017), which are characteristic of adolescence.

An increase of connectivity strength was also seen in the posterior cingulate node of the DMN in adolescents with higher stress levels. The same findings were previously associated to exposure to social stressors (Clemens et al., 2017), that could be associated to an enhancement of alertness state in the high stress group.

The between-group difference found in degree centrality of the visual central network in the high stress group emphasizes a higher number of edges connecting the visual central nodes to other brain regions (Soares et al., 2013). An increase of degree was seen in a sub-region of the posterior cingulate, which is part of the dorsal attention network, confirming the results of the connectivity strength analysis. In the Schaefer template, the posterior cingulate (together with the precuneus) is also part of the DMN, and a few studies suggested that sub-regions of the PCC are related to other networks such as attentional, somato-motor and executive control networks (Leech et al., 2012) (Veer et al., 2011). This might explain the relation between the structural changes in the PCC and stress-related responses. For example, in a previous study focused on PTSD, it was found that stress had implications beyond altering the connectivity of the posterior cingulum. Furthermore, they identified a direct correlation between the re-experiencing of PTSD symptoms and connectivity alterations (Weis et al., 2018). The authors proposed that alterations in connectivity were primarily associated to the real occurrence of neuropsychiatric symptoms associated with stress, rather than solely the stress factor itself. Other nodes emerging as hubs included those from the limbic, central executive, and attentional networks, aligning with findings from earlier research (Power et al., 2010, Sacchet et al., 2016).

The decrease of nodal betweenness centrality found in the left somato-motor network of the High stress could also be explained by morphological alterations during adolescence. With the onset of puberty, GM loss was seen firstly in primary sensorimotor areas and later in association areas (Gogtay et al., 2004, Ernst and Mueller, 2008) spreading rostrally over the frontal cortex and caudally over the parietal and temporal cortex (Paus, 2005). In developmental studies, somato-motor centrality measures were indicated as potential predictors of neuropsychiatric disorders, such as ADHD in children (dos Santos Siqueira et al., 2014).

Explorations into brain connectivity are impacted by methodological decisions encompassing factors like network segmentation, choice of neuroimaging technique, and the chosen parcelling approach. An important aspect in connectivity analyses is the selection of sparsity level, indicating the quantity of nodes and edges examined relative to the total potential connections in the graph. The decision to calculate graph theory metrics across seven different sparsity levels was made to ascertain whether statistically significant results were consistent across various levels, rather than being confined to a single level.

In a few studies the influence of sparsity level on the brain connectivity measures was investigated, by calculating the intra-class correlation coefficient (ICC) on both structural (DTI) (Dennis et al., 2012, Yuan et al., 2019) and rs-fMRI (Wang et al., 2011). For example, Dennis and colleagues found that LP and global efficiency were very unstable until sparsity level of 25%. Cluster coefficient had some dips within the sparsity range of 30–35%, whereas small-worldness presented many fluctuations, with peaks and dips (Dennis et al., 2012). Path length and global efficiency were less reliable than CP, since longer connections were seen to be trimmed before the shorter ones. Results on rs-fMRI confirmed CP low reliability, whereas nodal degree centrality showed high reliability (Wang et al., 2011). Finally, the most reliable connections were found to belong to the frontal cortex, suggesting the metric reliability was also influenced by the areas involved in the networks. Overall, this indicates the tight relationship between the calculation of graph theory measures and the sparsity level.

An important point to highlight in this study is the choice of events to measure the stress level for each participant. The Life Event Questionnaire is a self-reported psychological test (as an adaptation of the Stressful Life-Event Questionnaire by Newcomb et al., 1981) where each event was rated to have a negative or positive impact by the participants. LEQ had been previously used (Newcomb et al., 1981, Burt et al., 2016) to investigate the relationship between the brain structure and functioning and the capacity to cope with NLEs. For the same event, participants indicated if an event was making them feel happy or unhappy, showing an inter-subject variability in the event perception as positive or negative. While we selected only the negative scores from each question (i.e., events) based solely on the participant's ratings, this selection doesn't inherently classify the event as objectively stressful or otherwise. Furthermore, the absence of significant findings are unlikely to be influenced by differences in DAWBA scores, given the low mean averages observed in both groups. In addition to the computer based scores, clinical raters using the DAWBA data diagnosed 3 participants for anxiety disorder and 1 participant for major depression. Thus there is no significant presence of psychiatric disorders in this cohort – consistent with the fact that they were recruited from the community and thus were a ‘low-risk’ group.

One of the limitations of this study might be represented by the template choice to extract networks. While Yeo’s 400 parcellations gave a better understanding of where potential structural connectivity changes might occur, one potential disadvantage might be that parcellations included only the cortex, and none of the sub-cortical structures, such as amygdala, basal ganglia and thalamic nuclei, which are important to understanding what happens in the brain connectivity with stress. The use of parcellation based on adult brains for a sample of adolescents introduces a potential limitation in the generalizability and accuracy of our findings. The brains of adolescents undergo significant structural and functional changes during this developmental stage, and these changes may not be accurately represented by parcellation derived from adult brains.

In this study, the focus was on examining alterations in gray matter networks among healthy adolescents who encountered negative life events. The analysis involved individual-level brain network extraction from gray matter segmentations, shedding light on the structural connections between brain regions within the adolescent context. The results revealed differences within specific regions at a local scale, while no distinctions were evident in the broader graph theory metrics. This study findings offer potential insights into how stress can influence structural connectivity in adolescents, thereby contributing to a deeper understanding of the impact of environmental factors on typical development and the potential emergence of emotional and behavioral disorders.

## CRediT authorship contribution statement

Conceptualization: Sibilia and Bokde. Data Curation: Sibilia. Formal analysis: Sibilia. Investigation: Sibilia and Bokde. Methodology: Sibilia, Bokde and IMAGEN Consortium. Funding acquisition: Bokde. Project administration: Sibilia. Resources: Bokde and IMAGEN Consortium. Supervision: Bokde. Writing – original draft: Sibilia. Writing – review & editing: Sibilia and Bokde.

## Ethical statement

I have read and have abided by the statement of ethical standards for manuscripts submitted to IBRO Neuroscience Reports.

## Declaration of Competing Interest

Dr. Banaschewski has served as an advisor or consultant to Actelion, Hexal Pharma,Bristol-Myers Squibb, Desitin Arzneimittel, Eli Lilly, Lundbeck, Medice, Neurim Pharmaceuticals, Novartis, Pfizer, and Shire, UCB, and Vifor Pharma; he has received conference attendance support, conference support, or speaking fees from Eli Lilly, Janssen McNeil, Medice, Novartis, and Shire, and UCB; and he is involved in clinical trials conducted by Eli Lilly, Novartis, and Shire and Viforpharma; he received royalities from Hogrefe, Kohlhammer, CIP Medien, Oxford University Press; the present work is unrelated to these relationships. Dr. Barker has received honoraria from General Electric Healthcare for teaching on scanner programming courses and acts as a consultant for IXICO. Dr. Poustka served in an advisory or consultancy role for Roche and Viforpharm and received speaker’s fee by Shire. She received royalties from Hogrefe, Kohlhammer and Schattauer. The present work is unrelated to the above grants and relationships. The other authors report no biomedical financial interests or potential conflicts of interest.
