# Supplementary material for: The relationship between negative life events and cortical structural connectivity in adolescents
Source: IBRO Neurosci Rep. 2024 Feb 1;16:201–10. doi: 10.1016/j.ibneur.2024.01.012 (PMC10859284; doi:10.1016/j.ibneur.2024.01.012)
Supplement: Supplementary file 1 — Supplementary material [file mmc1.docx]

**Supplementary material**

**“THE EFFECT OF NEGATIVE LIFE EVENTS ON CORTICAL STRUCTURAL CONNECTIVITY IN ADOLESCENTS” by Sibilia et al.**

**Supplementary Methods**

*Atlas-based ROI approach*


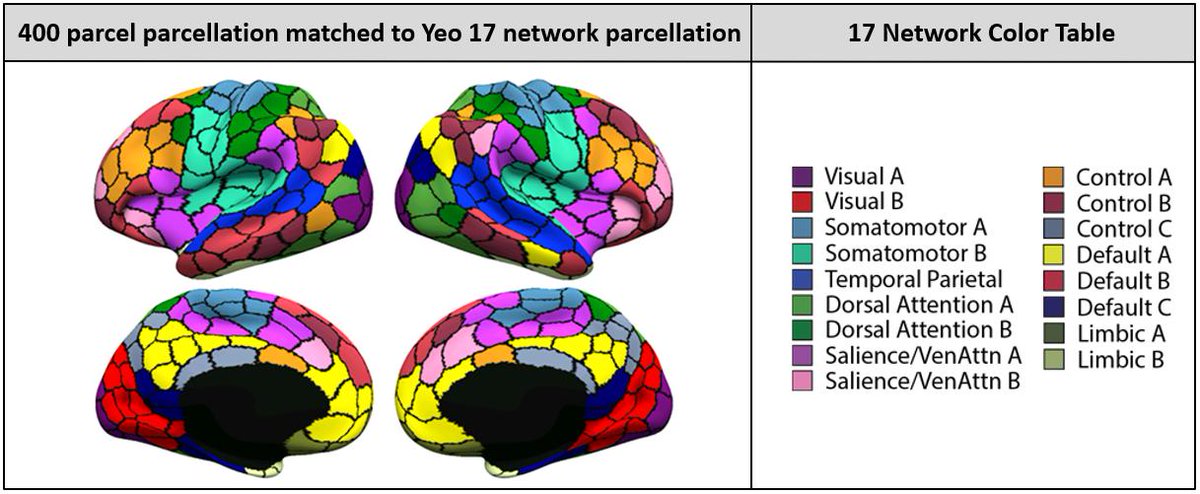


**Supplementary Figure 1**: Representation of the 400 parcel and 17 network parcellation used in this study to carry out an ROI-based analysis (Yeo et al., 2011).

To get the coordinates, we calculated the centroids for each parcel/ROI. To do so, we used the command: **3dcalc –prefix *output.nii* –a *input* –expr ‘within(a,nROI, nROI)’** in AFNI to extract each ROI from the brain parcellation singularly; afterwards, we used a command in FSL to calculate the coordinates of the centroid: **fslstats –t *output.nii* –C.**


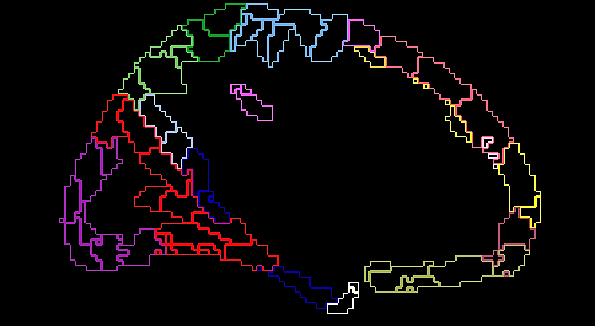


**Supplementary Figure 2**: Sagittal view of the single parcels that form the 17 networks. Different colors identify the networks according to the lookup table indicated by Schaefer et al., 2018. The list of all the ROI order is available at this webpage: <https://github.com/ThomasYeoLab/CBIG/blob/master/stable_projects/brain_parcellation/Schaefer2018_LocalGlobal/Parcellations/MNI/Schaefer2018_400Parcels_17Networks_order.txt>.


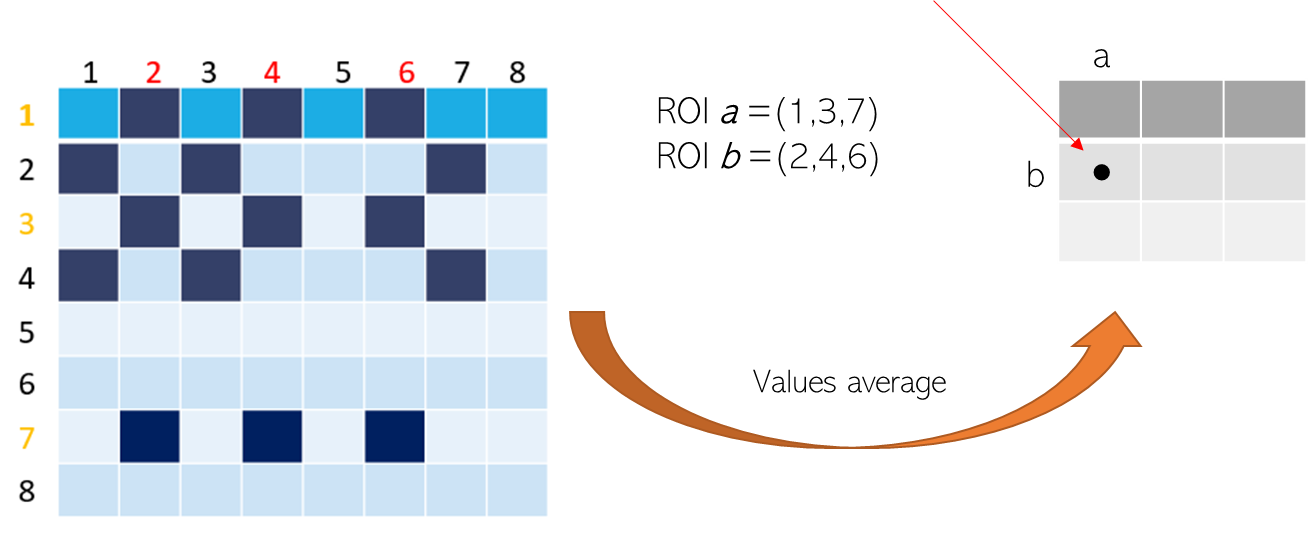


**Supplementary Figure 3**: Graphical representation of the correlation matrices reshape steps. The resize approach consisted in taking all the elements of the rows belonging to the “ROI” *a* and of the column belonging to the ROI *b*, calculate the average of all these values and store the new value in the position (a,b) of the reshaped matrix.

*Resize of the correlation matrices*

Supplementary Figure 3 describes the steps we did to resize the correlation matrices for each individual (with different number of nodes) in matrices of size 400x400 nodes (i.e. the number of parcels in Schaefer template). For illustrative purposes, the image below shows the averaging approach for each parcel. The first step was identifying the single ROIs (in this example ROI ***a*** and ROI ***b***) that belonged to the same parcel in the Schaefer atlas. We took all the elements (i.e. indices) of the rows belonging to the ROI ***a***, and all the elements of the columns belonging to the ROI ***b***, and calculated the average across all the values of the two ROIs. Finally, we stored the new value in position (***a,b***) of the resized matrix. This was done for all the 400 ROIs and for each participant.

**Supplementary Results**

*Results of partial linear correlation for the bigger group (N=976)*

*
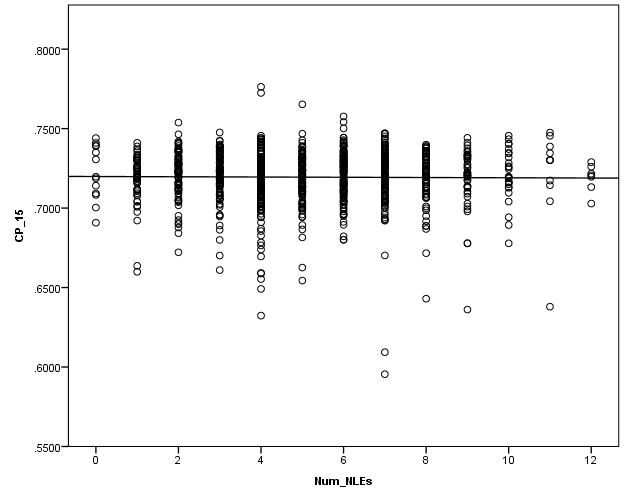
a)*

*
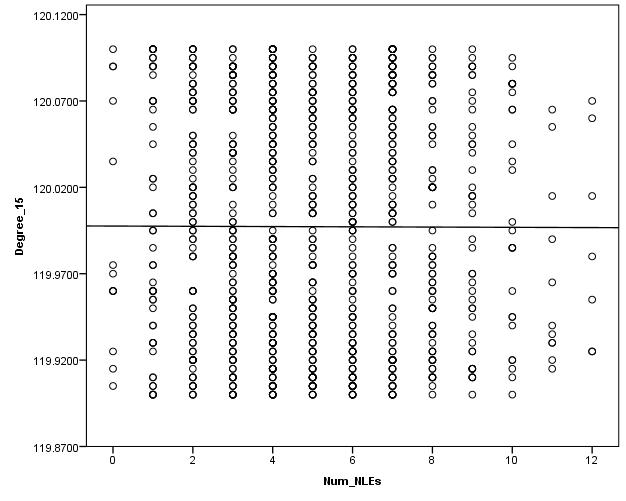
b)*

*
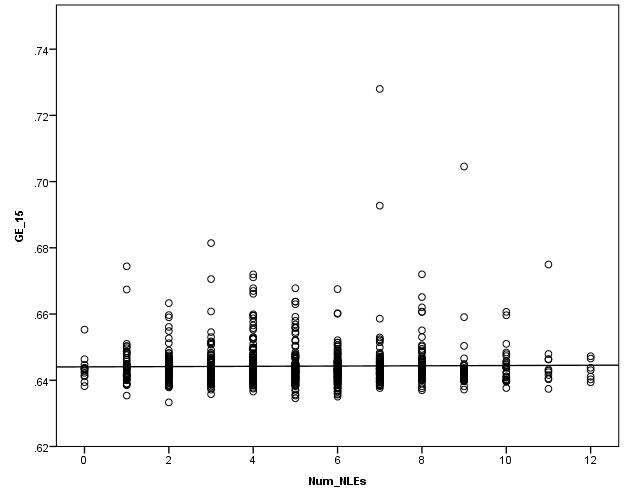
c)*

*
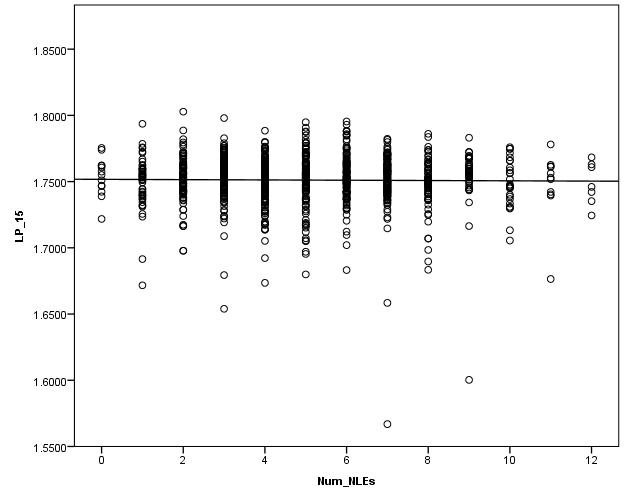
d)*

*
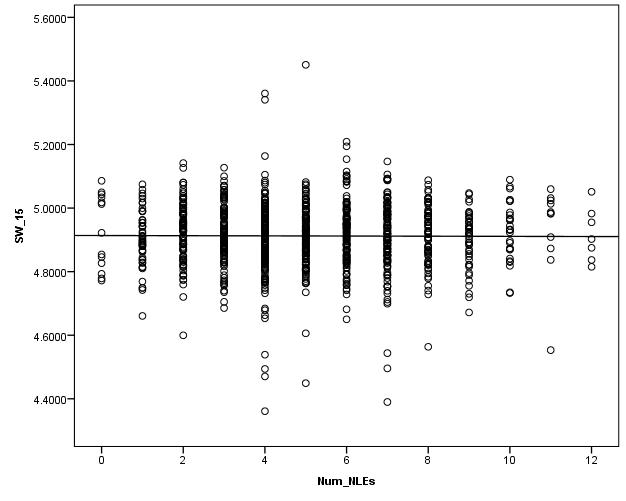
e)*

**Supplementary Figure 4:** Scatterplots representing the non-significant results of partial correlations between each graph theory measure and the level of stress, indicated by the total number of negative life events (NLEs) at sparsity 15%. Graph theory measures: a) CP = cluster coefficient (r =0.1536); b) Degree centrality (r = 0.13445); c) GE = global efficiency (r = 0.210); d) LP = path length (r = 0.1918); e) SW = small worldness (r = 0.1612).


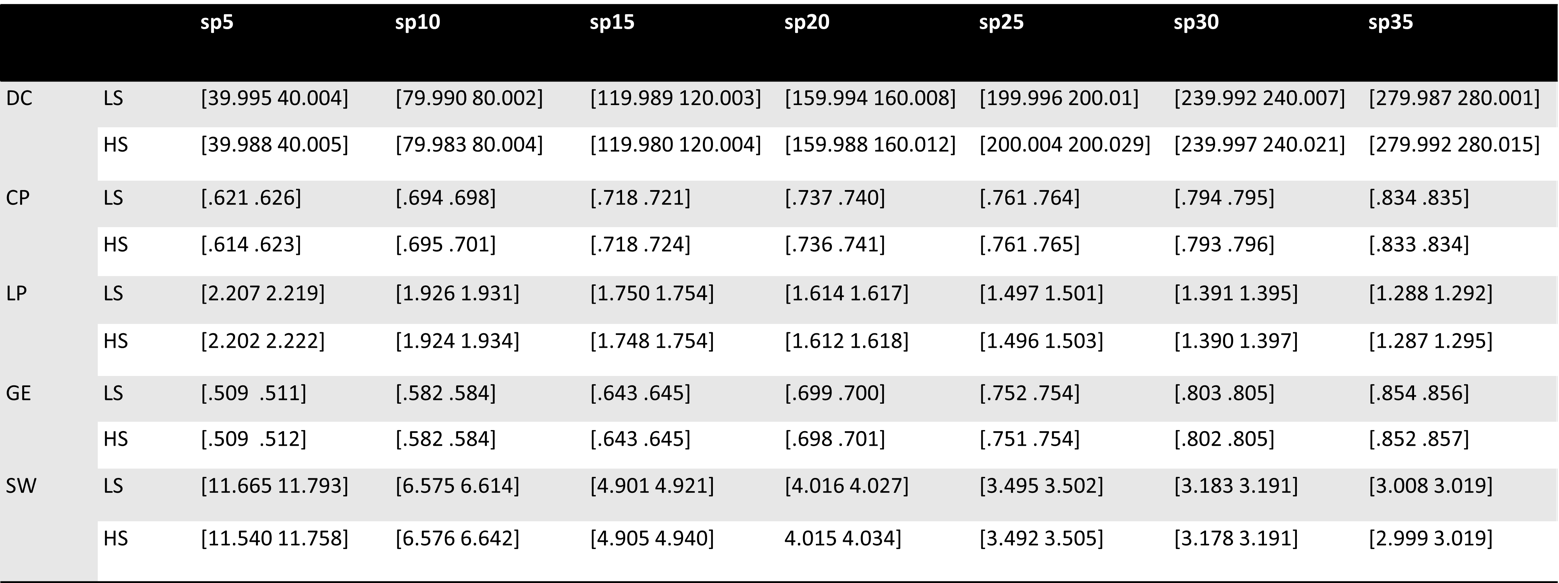


**Supplementary Figure 5**: 95% confident intervals (CI) values of global graph theory measure, which showed no significant differences between groups. Abbreviations: sp=sparsity, LS=Low Stress, HS=High Stress, DC= Degree Centrality, CP= Cluster Coefficient, LP=Path Length, GE=Global Efficiency, SW=Small Worldness.

**Supplementary discussion**

It has been shown that grey matter volume changes related to negative life events in adolescence are linked to the time of when such events happen, as well as the type of event they experience: Tyborowska and colleagues (2018) divided the event type into two groups, that is personal events (related to adolescents’ relationship with parents and themselves) and social ones (related to their relationships with peers) [1]. Researchers found that adolescents between 14 and 17 years old had lower GMV due to NLE, and that such reductions depended on the nature of the negative life experience: more personal early-life stressful events were associated with larger reductions in GMV over anterior prefrontal cortex, amygdala and insula; whereas ongoing stress from the adolescents' interactions with their peers was related to smaller reductions over the orbitofrontal lobe and anterior cingulate cortex, which are involved in emotional processing and reward system. Researchers suggest that early-life stress accelerates pubertal development, whereas a difficult social life disturbs brain maturation with potential mental health implications. Another study taking into account our same psychological test to measure the stress level in adolescents, divided the events in three groups, i.e. event related to family, to personal distress and accident, measuring the correlation between functional connectivity in limbic regions, finding correlation between the type of events and the neural response, specifically for the ‘distress’ sub-scale of stressful events [2]. This highlights the fact that the choice of NLEs grouping can affect connectivity analysis. In our study, we divided the two groups based on the total number of events each teenager experienced in their lifetime. It would be worthy looking at the same type of analysis with a different event grouping approach.

**Supplementary references**

1. Tyborowska, A., et al., *Early-life and pubertal stress differentially modulate grey matter development in human adolescents.* Sci Rep, 2018. **8**(1): p. 9201.

2. Burt, K.B., et al., *Structural brain correlates of adolescent resilience.* J Child Psychol Psychiatry, 2016. **57**(11): p. 1287-1296.
